# Consumers’ Knowledge, Attitudes, and Sensory Perception of Soilless-Grown Strawberries in the Context of Sustainable Diet

**DOI:** 10.3390/foods15101614

**Published:** 2026-05-07

**Authors:** Patrizia Calella, Mario Siervo, Concetta Paola Pelullo, Fabrizio Liguori, Giuliana Valerio, Giorgio Liguori, Francesca Gallè

**Affiliations:** 1 Saint Camillus International University of Health and Medical Sciences (UniCamillus), 00131 Rome, Italy; 2 Dementia Centre of Excellence, School of Population Health, Curtin University, Perth 6102, Australia; 3 Department of Medical, Movement and Wellbeing Sciences, University of Naples “Parthenope”, 80133 Naples, Italy

**Keywords:** sustainability, soilless cultivation, hydroponic systems, organic food, consumer perception, sensory analysis

## Abstract

The promise of sustainability and alternative agricultural methods to lessen the environmental impact of food production has drawn more attention in recent years. This study aimed to assess consumers’ knowledge and attitudes toward soilless cultivation, sustainable and organic foods, to identify socio-demographic correlates of these attitudes, and to evaluate the sensory perception of strawberries grown using traditional versus soilless methods. A cross-sectional survey was conducted among 298 adults in Southern Italy using an anonymous questionnaire assessing knowledge, attitudes, and purchasing behaviors related to sustainability and agricultural practices. A subsample of participants also took part in a blind sensory evaluation comparing soil-grown and soilless-grown strawberries. Results showed moderate awareness of sustainability and organic food concepts, but limited knowledge of soilless and hydroponic cultivation, with only 26% of participants correctly identifying all definitions. Sustainability and hydroponic production were rarely considered in food purchasing decisions. Higher educational attainment was associated with greater knowledge and a higher propensity to purchase sustainable and organic foods. Sensory analysis revealed a significant preference for soilless-grown strawberries in terms of odor, flavor, and overall sensory quality, while color and texture did not differ between cultivation methods. In conclusion, increasing consumer knowledge appears essential to enhance acceptance of innovative and sustainable agricultural systems.

## 1. Introduction

In recent years, the concept of sustainability has gained significant attention due to the pressing environmental and social challenges the planet is facing. The environmental impacts of human activities are not limited to climate change but also include soil erosion, deforestation, water pollution, biodiversity decline, and a range of interconnected issues [1]. The food sector is a major contributor to global greenhouse gas emissions, accounting for approximately one-third of total anthropogenic emissions [2]. Recent evidence indicates that agri-food systems are responsible for approximately one-third of total anthropogenic GHG emissions worldwide, encompassing emissions from agricultural production, land-use change, and the entire food supply chain, including processing, transportation, retail, and consumption [3]. According to the most recent FAO estimates, global agri-food system emissions reached approximately 16.5 gigatonnes of CO_2_-equivalent in 2023, showing a marked increase compared to previous decades and confirming a persistent upward trend driven by population growth, intensification of agricultural practices, and increasing demand for resource-intensive foods [4]. Although the relative contribution of the food system to total emissions has slightly decreased over time due to the expansion of other sectors, absolute emissions have continued to rise, highlighting the growing environmental burden associated with food production and consumption [5]. Emissions related to pre- and post-production activities, such as food processing, packaging, transportation, and retail, have increased substantially in recent years, reflecting the increasing complexity and globalization of food systems. These components have shown particularly rapid growth, with some estimates indicating increases of over 30% since the early 2000s, and even higher growth rates in certain sectors such as refrigeration and distribution. At the same time, agricultural production itself, particularly livestock and fertilizer use, remains a dominant source of methane and nitrous oxide emissions, both of which have a significantly higher global warming potential than carbon dioxide [5].

For this reason, sustainable methods have been recently developed to reduce the environmental impact of food production. Sustainable agriculture, in particular, aims to meet current food needs without compromising the ability of future generations to meet theirs, emphasizing practices that protect the environment, public health, human communities, and animal welfare [6]. In recent decades, global agricultural production has increased significantly due to improvements in crop yields and technological advancements, allowing food systems to support a growing population. For instance, global cereal production has more than tripled since the 1960s, largely driven by yield improvements rather than expansion of cultivated land [2,7].

However, despite these gains, ensuring food security remains a major global challenge. According to the Food and Agriculture Organization (FAO), food security is achieved when all people, at all times, have physical, social, and economic access to sufficient, safe, and nutritious food [8]. Current projections indicate that food production will need to increase by approximately 50–60% by 2050 to meet the demands of a growing global population, while simultaneously reducing environmental impacts [9,10].

Organic farming, a subset of sustainable agriculture, avoids the use of synthetic fertilizers and pesticides, promoting ecological balance and biodiversity [11]. Soilless cultivation systems, such as hydroponics, have emerged as innovative alternatives to traditional soil-based agriculture. Initially developed to combat soil-borne pathogens, these systems offer optimal control over plant growth conditions, leading to high productivity and product quality, as well as efficient use of water and fertilizers [12]. Despite these advantages, consumers often perceive soilless techniques as unnatural and associated with lower quality [13]. Consumers play a pivotal role in mitigating environmental impacts through their food choices [14]. However, evidence suggests that consumers’ understanding of “sustainability” in the context of the food supply chain remains limited, with price, taste, and health being the primary determinants of food choices rather than sustainability [15]. Nevertheless, significant gaps remain in consumers’ knowledge of specific sustainability topics, including the environmental impacts of their food choices [15].

Understanding consumer awareness and perceptions is crucial, as they can influence purchasing decisions and acceptance of products derived from alternative farming systems. In this study, we chose to focus particularly on berries, and more specifically on strawberries, as they represent a widely consumed fruit with high nutritional value and sensory appeal. Their popularity, seasonal availability, and susceptibility to different cultivation methods make strawberries an ideal model for assessing consumer responses to alternative agricultural systems. Strawberries are a high-value crop widely appreciated for their sensory attributes, including flavor, aroma, and texture. According to FAOSTAT data, over 10 million tonnes of strawberries are produced worldwide annually, with China, the United States, and several European nations being the top producers. Strawberries are one of the most important berry crops in terms of worldwide market demand and trade within the fresh fruit sector, and their economic significance is significant [16]. Strawberries are a particularly good model for examining the effects of novel agricultural systems on both product quality and consumer perception because of their high perishability, sensitivity to production conditions, and robust consumer demand.

The cultivation method can significantly influence these sensory properties. Studies comparing organic and conventional strawberry farming have evaluated differences in fruit quality, including mineral content, shelf life, and phytochemical composition, highlighting that organic strawberry farms produce higher quality fruit [17]. Similarly, preliminary research on strawberries grown in soilless systems has investigated their volatile profiles to assess potential differences in aroma compounds compared to soil-grown counterparts [18]. By growing plants in nutrient-rich water, hydroponics can reduce the environmental impact of food production while potentially enhancing the nutritional quality of produce [19]. Studies have shown that soilless cultivation can substantially reduce water use, with reported savings ranging from 40% to 90% depending on the system design, while enabling precise nutrient delivery through fertigation systems [20]. This approach improves nutrient use efficiency and can reduce fertilizer losses and nutrient leaching by up to 70% compared to conventional soil-based agriculture. Furthermore, these systems reduce soil degradation and can achieve higher yields per unit area, contributing to more resource-efficient and sustainable production [20,21]. However, consumer awareness and acceptability of hydroponically grown produce represent a research area that requires further investigation.

Food choices and perceptions of sustainable production systems are significantly influenced by consumer-related factors in addition to product-related attributes. Age, gender, and educational attainment are examples of sociodemographic factors that have been linked to variations in environmental consciousness, food knowledge, and purchase habits. Recent research shows that sociodemographic traits have a significant impact on consumer segmentation in sustainable food consumption, leading to a variety of behavioral patterns and preferences [22]. Systematic evidence also highlights that gender differences play a significant role, with women generally showing greater concern for sustainability, health, and ethical aspects of food, while men tend to exhibit less engagement in sustainable dietary practices [23]. Similarly, age and education have been identified as key determinants of consumer attitudes, knowledge levels, and openness to innovative food technologies, including organic and novel food products [24,25]. In a similar vein, consumer attitudes, knowledge, and receptivity to novel and organic food technology have been found to be significantly influenced by age and education [26]. Despite the growing body of evidence on consumer behavior toward sustainable and innovative food systems, further research is needed to better understand how these socio-demographic factors interact with specific production methods, such as soilless cultivation, and influence both attitudes and sensory perception.

Although previous studies have investigated consumer perceptions of sustainable and innovative food systems, most of the available evidence has focused on general attitudes toward sustainability or on single aspects such as organic food or hydroponic production. In addition, limited research has simultaneously explored the relationship between consumer knowledge, socio-demographic determinants, purchasing behaviors, and sensory perception within the same study framework.

To our knowledge, few studies have integrated both a survey-based assessment of knowledge and attitudes and a direct sensory evaluation of products derived from alternative agricultural systems. This combined approach is particularly relevant, as consumer acceptance is influenced not only by cognitive factors (e.g., knowledge and beliefs) but also by direct sensory experience.

Therefore, this study provides a comprehensive evaluation of consumers’ knowledge, attitudes, socio-demographic correlates, and sensory perception of soilless-grown strawberries, offering a more integrated perspective on the determinants of acceptance of sustainable and innovative agricultural practices.

Based on these premises, this study aimed to: (1) assess consumers’ knowledge and attitudes toward soilless cultivation, sustainable and organic foods; (2) investigate the role of socio-demographic factors (age, gender, educational level, and dietary habits) as potential determinants of these attitudes and purchasing behaviors; and (3) evaluate the sensory perception of strawberries grown using traditional versus soilless cultivation methods.

## 2. Materials and Methods

This study followed a cross-sectional design and involved workers from two secondary sector companies of the Campania region, Italy. Permission to distribute an anonymous questionnaire and to conduct a sensory test among the employees was requested from the company directors. An email was sent to the employees, explaining the objectives and methods of study. The email emphasized that participation would include completing an anonymous questionnaire and, where applicable, a second phase in which participants would taste two strawberry samples under blindfold conditions and answer a series of questions. Participation in the tasting phase was contingent on the absence of food allergies or other issues related to strawberry consumption. Importantly, participants were given the option to take part in the first phase of the study only, ensuring flexibility in participation.

The study sample included adult workers from two companies in the Campania region (Southern Italy). Participants covered a broad age range and included both males and females with varying educational levels. A detailed description of the socio-demographic characteristics of the sample is reported in the results section.

The first section of the questionnaire gathered general information about participants, including their age, gender and educational level. Additionally, participants were asked to report their current height (in meters) and weight (in kilograms) to calculate Body Mass Index (BMI). Dietary habits were also explored, including whether their diet was omnivorous, vegetarian, vegan, or other, as well as whether they were following a specific diet at the time of the study.

The second section included 10 custom-designed questions aimed at assessing participants’ knowledge and attitudes toward soilless agricultural products, as well as sustainable and organic foods. The questionnaire explored familiarity with key concepts (e.g., sustainability, organic food, soilless and hydroponic cultivation), the ability to correctly identify their definitions, and purchasing behaviors related to these attributes. Response formats included dichotomous (Yes/No), multiple-choice, and Likert-scale questions with five response options ranging from “Always” to “Never”. A detailed description of the questionnaire, including all items and response options, is provided in Supplementary Table S1.

The questionnaire was specifically designed for this study, drawing on and informed by existing literature on consumer perceptions of sustainable and innovative food systems. Prior to data collection, it was pilot-tested on a sample of 30 individuals to assess clarity, comprehensibility, and perceived difficulty of the items. Based on participants’ feedback, minor revisions were made to improve item wording and response options. This preliminary testing ensured adequate content and face validity of the instrument.

Finally, four questions assessing the consumption of red fruits, specifically focusing on the frequency and seasonal patterns of strawberry and berry consumption, were included. This addition was made in preparation for the subsequent sensory analysis on strawberries. People who agreed to participate in the sensory test were asked to sanitize their hands before tasting. Two strawberries, one grown using traditional methods and the other grown using soilless methods, were presented to participants. Only the experimenter was aware of the origin of each fruit. Participants were asked to taste one sample at a time and respond to sensory questions. These questions were aimed at assessing the color, odor, taste, and texture of each strawberry using a 5-point hedonic Likert scale with options ranging from “I like it very much” to “I don’t like it at all.” In addition to the Likert scale, participants were asked to freely provide descriptive adjectives for each sensory attribute to capture qualitative aspects of sensory perception and differences between the two types of strawberries.

The sensory questionnaire was adapted from a validated format [27] with modifications to include free-choice descriptors for each sensory attribute. This approach aimed to identify potential differences in consumers’ perceptions of traditional and soilless-grown strawberries.

### Statistics

Descriptive statistics were used to summarize the demographic characteristics of the participants, as well as their dietary habits and knowledge of agricultural practices and sustainability. Means and standard deviations were calculated for continuous variables, while frequencies and percentages were reported for categorical variables. Skewness and kurtosis values were used to assess the value distribution for each variable.

Due to the ordinal nature and non-normal distribution of the variable, the relationships between knowledge and purchasing behaviors related to soilless agricultural products, sustainable and organic food were assessed using Spearman’s correlation analysis.

Furthermore, multiple logistic regression analyses were performed to identify possible predictors and correlates of the propensity to purchase hydroponic cultivated foods, sustainable and organic products (1 = never or rarely, 2 = occasionally or often). Independent variables entered in the model were those which showed significant associations in the correlation analysis: educational level (1 = non-graduates, 2 = graduates or postgraduates) and the correct knowledge of the meaning of the terms hydroponic, sustainable and organic (1 = yes, 2 = no). Gender (0 = male, 1 = female) and age (1 = lower than median value, 2 = higher than median value) were also included in the models. Odds ratios (ORs) with 95% confidence intervals (95%CI) were reported to quantify these associations.

For sensory analysis, paired *t*-tests were conducted to compare participants’ sensory evaluations (color, odor, taste, and texture) of strawberries grown using traditional versus soilless methods, considering their normal distribution. Qualitative data from the free-choice descriptive adjectives were analyzed to identify recurring themes and capture nuanced differences in sensory perceptions. Data visualization techniques, such as bar plots and scatter plots, were employed to support the interpretation of results.

All statistical analyses were performed using the SPSS Statistics software, v. 28 (IBM Corporation, New York, NY, USA), with a significance level set at *p* < 0.05.

## 3. Results

A total of 298 participants completed the questionnaire, while 139 of them participated in the sensory analysis. The statistical power of the study was 0.45 for a moderate effect size (r = −0.11) for two groups with a significance level of 0.05 and a sample size of 139 [28]. The survey was completed by 298 people, whose ages ranged from 19 to 68 years old, with an average age of 45.2 ± 13.5 years. There were 175 females (59%) and 123 males (41%) in the sample. In terms of educational attainment, 34% of participants had a university degree, 45% had a high school diploma, and 21% had just completed elementary or middle school. Eighty-seven percent of participants said they were omnivores, 3% reported vegetarian or vegan diet. Furthermore, 17% of the sample said they presently adhere to a prescribed diet.

### 3.1. Knowledge and Attitudes Assessment

Moving on to the section of the questionnaire that assessed knowledge about hydroponic cultivation sustainability and organic products, Table 1 provides a detailed overview of the data.

Only 81 individuals, representing 26% of the entire sample, identified the correct definitions for sustainability, organic food, and hydroponic cultivation.

Based on the frequency distributions, food sustainability was generally not a major criterion in participants’ purchasing decisions. Specifically, 171 participants (57.4%) reported that they never select food based on sustainability, while 116 participants (38.9%) indicated doing so rarely or moderately (21.8% rarely; 17.1% moderately). Only 11 participants (3.7%) stated that they often consider sustainability when purchasing food.

Regarding organic food, 115 participants (38.6%) reported that they never purchase food because they are organic. A larger proportion, 157 participants (52.7%), stated that they consider the organic attribute rarely or moderately (36.6% rarely; 16.1% moderately). In addition, 26 participants (8.7%) reported that they often base their purchase decisions on whether a product is organic.

Finally, none of the respondents reported choosing foods based on whether they were produced with soilless or hydroponic systems, as all participants (100%) selected “never” for this item.

Spearman’s correlation analysis showed that correct knowledge was positively associated with both the propensity to purchase sustainable (ρ = 0.384, *p* < 0.001) and organic foods (ρ = 0.378, *p* < 0.001). Educational attainment was significantly associated with knowledge (ρ = 0.198, *p* < 0.001), but not directly with purchasing behaviors (Table 2).

To further investigate the factors influencing consumer choices, two separate binary logistic regression analyses were conducted. The first model (Table 3) examined predictors of the propensity to purchase sustainable food products, while the second model (Table 4) assessed predictors of the propensity to purchase organic food. In both models, age, gender, educational attainment, and relevant knowledge (about organic food or sustainability) were included as explanatory variables.

Due to the lack of variability in responses, it was not possible to perform a multiple logistic regression analysis for the variable assessing the propensity to purchase products based on hydroponic cultivation. In fact, the entire sample declared that they never consider hydroponic cultivation as a criterion when purchasing food products.

In both models, knowledge was positively related to propensity to purchase sustainable or organic foods.

### 3.2. Sensory Analysis

In the whole sample, a total of 139 subjects (60% women, average age of 47.4 ± 10.9 years) participated in the sensory analysis. In this group, approximately 77% stated that they usually consumed strawberries during their natural growth season between 1 and 5 times a week. When asked to indicate which period they consume strawberries, 48% responded spring, 42% spring and summer, 7% summer only, and the remaining part all year round. Regarding the consumption of berries such as blueberries, currants, and raspberries, 40% reported never consuming them, 45% occasionally, and the others frequently.

Table 5 shows the comparison between the sensory perceptions of strawberries produced using the two cultivation methods.

**Figure 1:**
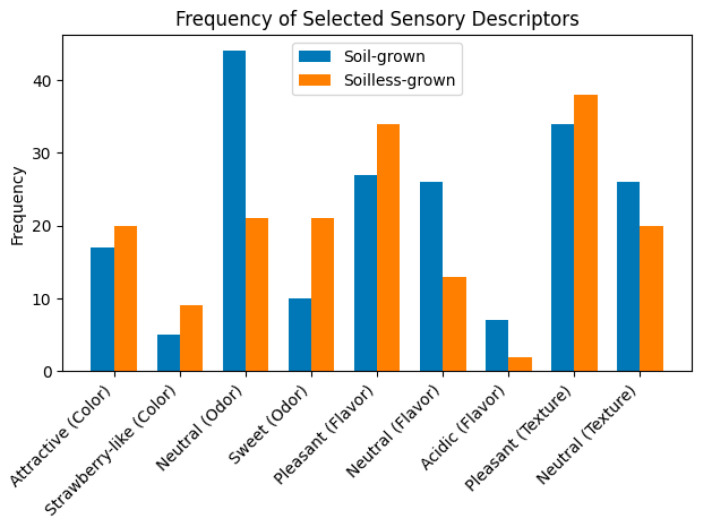
Frequency of main sensory descriptors reported by participants for soil-grown and soilless-grown strawberries.

As shown in Figure 1,soilless-grown strawberries were generally preferred over soil-grown strawberries, particularly in terms of odor, flavor, and overall sensory quality. However, differences in color and texture were not significant, indicating that these attributes were perceived as similar regardless of the cultivation method. The frequency of sensory descriptors reported by participants is detailed in Supplementary Table S2.

Participants frequently used adjectives such as red, bright, beautiful, and strawberry-colored. These descriptors emphasize a vivid and appealing appearance, with a strong inclination towards vibrant, intense red tones. Terms like fire, ruby and ripe also suggest a rich and mature color that is generally associated with visually appealing strawberries. The adjectives used indicate that both types of strawberries are perceived similarly in terms of color, with common descriptors emphasizing brightness, attractiveness, and vivid red tones.

Furthermore, regarding the odor perception, participants commonly used adjectives such as neutral, strawberry, sweet, fragrant and fruity. Notably, “sweet” was one of the most recurrent descriptors, for the soilless-grown strawberries suggesting that the odor was perceived as pleasantly; additionally, terms like light, fresh, and delicate were also commonly used, indicating a perception of a softer, more delicate scent compared to soil-grown strawberries.

To the flavor description, both soil-grown and soilless-grown strawberries were described using similar adjectives, with a strong emphasis on juiciness, sweetness, and a recognizable strawberry flavor. However, the soilless-grown strawberries received more frequent positive descriptors such as balance and full which could indicate a slightly more well-rounded flavor profile. The recurring use of juicy and sweet in both categories points to a shared perception of juiciness and sweetness that is appreciated regardless of cultivation method.

For the texture, both soil-grown and soilless-grown strawberries were described using similar terms, reflecting a generally positive perception of texture with a preference for softness and pulpiness. However, soilless-grown strawberries seemed to have a slightly more consistent texture. The repeated use of terms like “soft” and “velvety” for both types suggests a shared appreciation for smooth and soft textures.

## 4. Discussion

This study aimed to assess knowledge of soilless agricultural practices and sustainable, organic food and to evaluate sensory perceptions of strawberries grown through traditional and soilless cultivation methods in a sample of Italian adults. The findings reveal a moderate level of awareness regarding sustainability and organic farming, but a notably lower understanding of soilless and hydroponic cultivation. Only a quarter of participants correctly identified the definitions for all three agricultural practices, indicating a significant knowledge gap that could influence consumer choices and acceptance of alternative farming methods.

Consistent with the findings of our study, several investigations have highlighted a generally moderate level of awareness concerning sustainability and organic agriculture, while knowledge of soilless and hydroponic systems remains considerably limited. Similarly, Fernqvist et al. emphasized that while consumers show increasing interest in sustainable food choices, their actual knowledge remains superficial and does not always translate into consistent purchasing behaviors [23]. In line with these findings, Schäufele-Elbers and Janssen identified substantial heterogeneity in consumer awareness and attitudes toward sustainable food consumption, with knowledge levels varying significantly across population subgroups [22]. Moreover, recent evidence from Gallè et al. in an Italian sample confirmed that awareness and acceptance of innovative and sustainable food systems are still limited, particularly when unfamiliar production methods are involved, and are strongly influenced by socio-demographic factors such as education and age [24].

A systematic review synthesizing data from 56 studies found that consumer perceptions of soilless farming systems are influenced by product characteristics, as well as socio-cultural and psychological factors [13]. Sensory properties, sustainability, growing conditions, income, education, consumer knowledge, technology neophobia, and affinity were identified as significant factors affecting consumer views. The authors recommended that food industry and policymakers enhance consumer education about the characteristics and advantages of soilless farming systems to potentially increase purchase intentions toward these products [13].

These findings are consistent with previous studies showing that novel food technologies, including hydroponics and vertical farming, are often perceived as less natural compared to traditional agricultural methods, which may negatively influence acceptance despite their potential environmental benefits [29,30,31].

Recent research underscores the knowledge gap that persists in this domain. For instance, Spendrup et al. investigated consumer attitudes toward hydroponically grown vegetables, highlighting that fewer than half of respondents were familiar with hydroponic systems. Interestingly, belief in climate change and environmental awareness were associated with a higher willingness to consume such products, particularly when fertilizers derived from food waste were used [32]. Similarly, Califano et al. explored consumer perceptions of innovative cultivation methods for fresh herbs, including hydroponics. Their findings showed a preference for urban farming over hydroponics, with robotic farming being the least favored. Moreover, the study demonstrated that food technology neophobia negatively influenced acceptance of both hydroponic and robotic farming methods [33]. These results emphasize the importance of consumer education and targeted communication strategies to improve acceptance of alternative farming systems. In this context, our findings contribute to the growing body of evidence that increased knowledge about sustainable and innovative agricultural methods may enhance consumer openness and positively affect the marketability of sustainable products.

Beyond product characteristics, consumer-related factors appear to play a relevant role in shaping perceptions and acceptance of innovative and sustainable food systems. The observed correlations between age, educational attainment, and knowledge of sustainable practices suggest that demographic factors play a role in consumer awareness and behavior. Older and more educated individuals demonstrated higher levels of knowledge and a greater propensity to purchase sustainable and organic foods. These findings are consistent with existing literature indicating that education and age are significant predictors of environmentally conscious food choices [13].

In particular, higher educational attainment has been consistently associated with greater environmental literacy and openness toward food innovations, including alternative agricultural system [26,34].

In the present study, the sensory analysis demonstrated a general participants’ preference for soilless-grown strawberries over soil-grown counterparts, particularly concerning odor, flavor, and overall quality. These results align with previous studies suggesting that hydroponically grown strawberries can match or even surpass soil-grown varieties in sensory attributes. For instance, the study by Unal et al. demonstrated that soilless cultivation significantly improves pomological traits—such as fruit weight, firmness, and soluble solids content—especially in the ‘Camarosa’ strawberry variety. These findings suggest that hydroponic systems may enhance the commercial quality of strawberries compared to traditional soil-based cultivation [35].

More broadly, previous research has shown that controlled-environment agriculture, including hydroponic systems, allows precise regulation of water, nutrients, and environmental conditions, which may enhance product quality and uniformity [20]. This level of control may also affect volatile composition and other quality-related traits relevant to flavor and aroma, as shown in strawberries cultivated in soilless system [18]. Furthermore, the reduced variability associated with soilless systems may contribute to greater consistency in sensory attributes, which is a key factor in consumer satisfaction and acceptance.

Despite the valuable insights generated, this study presents several limitations that should be acknowledged. First, the sample was based on voluntary participation and convenience sampling within a limited geographical area, which may restrict the generalizability of the findings to the broader population. Then, the cross-sectional design did not allow us to establish causal relationships. In addition, data on dietary habits and anthropometric measures were self-reported and may therefore be subject to reporting bias. Moreover, the assessment of knowledge was based on multiple-choice questions, which may not fully capture the complexity of participants’ understanding of sustainability, organic food, and soilless cultivation. Lastly, the sensory analysis was conducted under semi-controlled conditions, and factors such as prior familiarity with strawberries or personal taste preferences could have influenced responses.

From a public health and sustainability perspective, improving consumer knowledge represents a key strategy to support the transition toward more sustainable food systems. Evidence suggests that targeted educational interventions, transparent labeling, and effective communication strategies can significantly improve consumer understanding and acceptance of sustainable and innovative food [15,36]. In addition, integrating sustainability into dietary guidelines and public health policies may contribute to enhancing food-related health literacy and promoting more sustainable dietary patterns [37].

However, considering the urgent need to turn towards sustainable productions, this study represents a contribution to the characterization of people’s propensity to consume sustainable products and their determinants. Further research in this field, involving wider and more representative samples, is needed to confirm our findings.

## 5. Conclusions

In conclusion, our findings show that, while soilless cultivation methods can produce strawberries with good sensory qualities, consumers’ awareness and understanding of these practices should increase for broader market acceptance. These findings emphasise the necessity of focused educational and communication initiatives meant to raise consumer awareness of innovative and sustainable agriculture practices. Public health authorities and policymakers should promote clearer and more accessible information on the environmental and nutritional benefits of soilless and sustainable production methods. In order to improve customer trust and enable informed food choices, producers and retailers, among other stakeholders in the food industry, should also play a significant role by implementing clear labelling and communication strategies.

Overall, integrating consumer education with policy interventions may support the transition toward more sustainable food systems and encourage the acceptance of alternative agricultural technologies. In this context, it is essential to promote clear, accessible, and evidence-based communication strategies that enable consumers to understand the differences between production methods and their implications for health and the environment. Enhancing food-related health literacy may empower individuals to make more informed and sustainable dietary choices.

## Figures and Tables

**Table 1:** Awareness and understanding of sustainability, organic food, and soilless cultivation in the sample.

	Total Sample (N = 298)
Do you know what is meant by soilless cultivation? (n/%Yes)	133/44.6
Do you know what is meant by hydroponic cultivation? (n/%Yes)	128/43
Detailed overview of the different answer options defining hydroponic cultivation (n/%)	
1. Cultivation of fruits only in water.	5/1.5
2. Cultivation of vegetables only in aqueous solutions.	94/31.8
3. Soilless cultivation using inorganic substrates.	79/26.4
4. Growing plants without soil, using mineral nutrient solutions in an aqueous solvent. (correct option)	120/40.3
Do you know what sustainability means? (%Yes)	139/46.6
Detailed overview of the different answer options defining sustainability (n/%)	
1. Exclusive protection of the environment	94/31.5
2. Meeting the needs of the present generation without compromising the ability of future generations to meet their own needs (correct option)	164/55.0
3. Meeting human needs without causing environmental pollution	36/12.2
4. Supporting human capabilities in cultivating the soil	4/1.3
Do you know what organic food means? (%Yes)	213/71.5
Detailed overview of the different answer options defining organic food	
1. Any product, of plant or animal origin, obtained through a process that involves the complete absence of pesticides, chemical fertilizers, and antibiotics—that is, external elements beyond what nature provides. (correct option)	185/62.1
2. Any product, exclusively of plant origin, obtained through a process that involves the complete absence of pesticides and chemical fertilizers.	97/32.4
3. Any product, exclusively of animal origin, obtained through a process that involves the complete absence of antibiotics.	16/5.5
4. Any product, of plant or animal origin, obtained through a process that involves the use of pesticides and chemical fertilizers but not antibiotics.	0

**Table 2:** Spearman correlation coefficients between educational attainment, knowledge, and purchasing behaviors.

Variable	1	2	3	4
1. Educational attainment	1.000	−0.003	−0.027	0.198 **
2. Propensity to purchase sustainable foods	−0.003	1.000	0.755 **	0.384 **
3. Propensity to purchase organic foods	−0.027	0.755 **	1.000	0.378 **
4. Correct knowledge of sustainability, organic food, and hydroponic cultivation	0.198 **	0.384 **	0.378 **	1.000

Values are Spearman’s rho coefficients. ** *p* < 0.01 (two-tailed).

**Table 3:** Results of the logistic regression analysis with propensity to purchase sustainable foods as dependent variable.

Explanatory Variables	Odds Ratio	95% Confidence Interval		*p* Value
		Lower Limit	Upper Limit	
Age	1.293	0.725	2.305	0.385
Gender	0.653	0.369	1.156	0.144
Educational attainment	1.380	0.751	2.535	0.299
Dietary habits	1.320	0.510	3.415	0.567
Knowledge about organic foods	44.912	6.111	330.085	<0.001

**Table 4:** Results of the logistic regression analysis with propensity to purchase organic foods as dependent variable.

Explanatory Variables	Odds Ratio	95% Confidence Interval		*p* Value
		Lower Limit	Upper Limit	
Age	1.321	0.731	2.389	0.357
Gender	0.803	0.445	1.450	0.467
Educational attainment	1.228	1.104	2.266	0.512
Dietary habits	1.179	0.506	2.745	0.703
Knowledge about sustainability	4.023	2.155	7.530	<0.001

**Table 5:** Sensory scores regarding soil- and soilless-grown strawberries.

Variable	Soil-Grown Strawberries	Soilless-Grown Strawberries	*p*-Value
Color	3.9 ± 0.6	4.0 ± 0.5	0.133
Odor	3.7 ± 0.6	3.9 ± 0.5	0.007
Flavor	3.9 ± 0.7	4.1 ± 0.6	0.025
Texture	3.7 ± 0.6	3.7 ± 0.6	0.919
Total sensory score	3.8 ± 0.5	3.9 ± 0.4	0.03

## Data Availability

The original contributions presented in the study are included in the article/Supplementary Material, further inquiries can be directed to the corresponding authors.

## Supplementary Materials

The following supporting information can be downloaded at: https://www.mdpi.com/article/10.3390/foods15101614/s1, Table S1: Questionnaire items assessing knowledge, attitudes, and purchasing behaviors related to sustainability, organic food, and soilless cultivation; Table S2: Frequency of Sensory Adjective Used by Participants After Sensory Testing of Soil- and Soilless-Grown Strawberries.
