# Supplementary material for: Consumers’ Knowledge, Attitudes, and Sensory Perception of Soilless-Grown Strawberries in the Context of Sustainable Diet
Source: Foods. 2026 May 7;15(10):1614. doi: 10.3390/foods15101614 (PMC13205151; doi:10.3390/foods15101614)
Supplement: Supplementary file 1 [file foods-15-01614-s001.zip › foods-4226625-supplementary.pdf]

Table S1. Questionnaire items assessing knowledge, attitudes, and purchasing behaviors related to sustainability, organic food, and soilless cultivation

Section: Sustainability

1. Do you know what sustainability means?

- Yes
- No

2. Do you choose food products based on their sustainability?

- Always
- Often
- Sometimes
- Rarely
- Never

3. What is meant by sustainability?

- Exclusive protection of the environment
- Meeting the needs of the present generation without compromising the ability of future generations to meet their own needs *(correct answer)*
- Meeting human needs without causing environmental pollution
- Supporting human ability to cultivate the soil

Section: Organic food

4. Do you know what organic food means?

- Yes
- No

5. Do you choose food products based on whether they are organic?

- Always
- Often
- Sometimes
- Rarely
- Never

6. What is meant by organic food?

- Any product of plant or animal origin obtained through a process that excludes pesticides, chemical fertilizers, and antibiotics *(correct answer)*
- Any product exclusively of plant origin obtained without pesticides and chemical fertilizers
- Any product exclusively of animal origin obtained without antibiotics
- Any product obtained using pesticides and fertilizers but not antibiotics

---

Section: Soilless and hydroponic cultivation

7. Do you know what soilless cultivation means?

- Yes
- No

8. Do you know what hydroponic cultivation means?

- Yes
- No

9. Do you choose products based on whether they are produced using soilless or hydroponic methods?

- Always
- Often

- Sometimes
- Rarely
- Never

10. What is meant by hydroponic cultivation?

- Cultivation of fruits only in water
- Cultivation of vegetables only in aqueous solutions
- Soilless cultivation using inorganic substrates
- Growing plants without soil using nutrient solutions in a liquid medium *(correct answer)*

Table S2. Frequency of Sensory Adjective Used by Participants After Sensory Testing of Soil- and Soilless-Grown Strawberries.

Descriptor group	Original adjectives	Soil-grown (n)	Soilless-grown (n)
**Color**			
**Red shades**	red, bright red,light red, strawberry red, fire red, vivid red, crimson, ruby, forest red	53	53
**Bright**	bright, shiny, fire	26	27
**Attractive**	beautiful, vivid, velvety	17	20
**Soft/Pale**	light, shaded	11	10
**Strawberry-like**	strawberry, strawberry-colored, strawberry and light	5	9
**Mature/Natural**	ripe, natural	6	7
**Normal**	normal	12	5
**Others**	dotted, spotted, glossy, silky, dark, homogeneous, slightly unripe, white-red	9	8
**Odor**			
**Neutral/None**	neutral, none, can't smell, don't know, normal	44	21
**Strawberry-like**	strawberry, strawberry-like	17	23
**Other**	mouth-watering, floral, faint, rounded, pungent, sour	12	16
**Pleasant**	pleasant, delicious, intense, rich	11	14
**Fruity**	fruity, fruit, fresh fruit	11	13
**Delicate**	delicate, light, mild	8	5
**Sweet**	sweet, sugary, honeyed	10	21
**Fresh**	Fresh	7	7
**Natural**	natural, nature, wood, earth	6	8
**No adjective provided**		13	10
**Flavor**			
**Pleasant**	pleasant, very good, good, velvety, balanced	27	34
**Neutral/None**	neutral, normal, none, don't know	26	13
**Juicy**	Juicy	22	22
**Sweet**	sweet, sugary, sweet and sour, rich	21	24
**Fruity/Fresh**	fruit, fresh, fruity	17	15
**Strawberry-like**	Strawberry	16	25
**Acidic/Sour**	sour, tart, slightly unripe	7	2
**Other**	watery, strong, rounded, enveloping, pungent, full	5	4
**Consistency**			
**Pleasant**	pleasant, good, excellent, ok, velvety, distinctive	34	38
**Normal**	Normal	26	28
**Neutral/None**	neutral, none, don't know, seedy	26	20
**Soft**	soft, tender, crunchy and soft, smooth, soft and seedy, like strawberry	18	18
**Firm**	firm, hard, compact, tough, dense, consistent	12	11
**Pulpy**	pulpy, plumo, fleshy	8	8
**Other**	crunchy, crunch, grainy, uneven, round, with seeds, strange	6	6
**No adjective provided**		9	10
